# Supplementary material for: Identification of a Munc13-sensitive step in chromaffin cell large dense-core vesicle exocytosis
Source: eLife. 2015 Nov 17;4:e10635. doi: 10.7554/eLife.10635 (PMC4798968; doi:10.7554/eLife.10635)
Supplement: Source code 1. — Macro for kinetic analysis of chromaffin cell capacitance traces developed with Igor Pro v6.32A (WaveMetrics) and Patcher’s Power Tools v2.19 (http://www3.mpibpc.mpg.de/groups/neher/index.php?page=software). Capacitance changes were triggered with calcium uncaging and the traces recorded with PatchMaster v2.20 (HEKA). DOI: http://dx.doi.org/10.7554/eLife.10635.017 [file elife-10635-code1.zip › Three_Exponential_Fit_Macro_Igor.rtf]

#pragma rtGlobals=1		// Use modern global access method.Macro TriFitFlash(wb,FitStartSt,FitEndSt,DBExp, weight)								//This macro allows to fit triple exp function to waves while reading them from Pulse filestring wb="Group_name";variable FitStartSt=0.46992,FitEndSt=6.58,DBExp=3,weight=10prompt wb,"Input base wave name"prompt FitStartSt,"Input flash time"prompt FitEndSt,"Input end time for fitting"prompt DBExp,"Double or triple exponential fit? 2: Double; 3: Triple"prompt weight,"Input weighting factor for fit"string FileName,wavebase,CmN,FitCmN,AmpN,CaN,TempFitCmN,CaNcorr,caname0,caname1,caname2,RN,Rt,grnam,xname,wavn,F1n,F2n,F3n,tempvariable fn,i,j,w=550,h=200,modi,k,Fur_exist,t_startExtr,Dfit0variable /G t_start=-1, ExPnts=0variable Pe=0.2,Rmin,Rmax,Keff,min,max,Cm_startstring MaxRaten,FitSN,FitEN,A0n,A1n,tao1n,A2n,tao2n,A3n,Tao3n,Amp1n,Amp2n,Amp4n,commentn,Fitstartn,Fitendsn,FileNameNstring DelayN,IntAmp1N,IntAmp2N,IntAmp4N,cellN,DiffRa,SeriesN,RateSusN,weightingNvariable/G V_FitMaxIters=400,fn_G,axislow,axishigh,FitEndvariable InitialFitOKflag		|MyInitialize()	silent 1	Make/o/n=100 gr_index,ser_index,sw_index	// 	Load group numbers:			gr_index={}// 	Load series numbers:			ser_index={}		//	Load sweep numbers							sw_index={} 	newpath /o /m="select folder with data file" liPath	PathInfo liPath		FitSN="FitSN"+wb	FitEN="FitEN"+wb	A0n="A0n"+wb	A1n="A1n"+wb	A2n="A2n"+wb	A3n="A3n"+wb	tao1n="tao1n"+wb	tao2n="tao2n"+wb	tao3n="tao3n"+wb	Amp1n="Amp1n"+wb	Amp2n="Amp2n"+wb	Amp4n="Amp4n"+wb	caname0="Ca0"+wb	caname1="bextraCa0"+wb	caname2="Ca2"+wb	commentn="Comment"+wb	Fitstartn="Fitstart"+wb	Fitendsn="Fitends"+wb	DelayN="Delay"+wb	cellN="cellN"+wb	SeriesN="SeriesN"+wb	RateSusN="RateSus"+wb	FileNameN="FileName"+wb	WeightingN="Weighting"+wbExPnts=0If(Waveexists($A0n)==1)	DoAlert 1, "A set of analyzed data exists - append?"		if(V_Flag==1)		ExPnts=numpnts($A0n)		Redimension /n=(ExPnts+numpnts(gr_index))	$A0n,$A1n,$tao1n,$A2n,$tao2n,$A3n,$tao3n,$DelayN, $RateSusN, $WeightingN		Redimension /n=(ExPnts+numpnts(gr_index))	$Fitstartn,$Fitendsn,$cellN,$SeriesN,$Amp1n,$Amp2n,$Amp4n		Redimension /n=(ExPnts+numpnts(gr_index)) 	$commentn, $FileNameN	else		make/n=(numpnts(gr_index))/o	$A0n,$A1n,$tao1n,$A2n,$tao2n,$A3n,$tao3n,$DelayN, $RateSusN, $WeightingN		make/n=(numpnts(gr_index))/o	$Fitstartn,$Fitendsn,$cellN,$SeriesN,$Amp1n,$Amp2n,$Amp4n		make/n=(numpnts(gr_index))/t/o $commentn, $FileNameN	endifelse 		make/n=(numpnts(gr_index))/o	$A0n,$A1n,$tao1n,$A2n,$tao2n,$A3n,$tao3n,$DelayN, $RateSusN, $WeightingN		make/n=(numpnts(gr_index))/o	$Fitstartn,$Fitendsn,$cellN,$SeriesN,$Amp1n,$Amp2n,$Amp4n		make/n=(numpnts(gr_index))/t/o $commentn, $FileNameNendif	Edit $FileNameN,$cellN,$SeriesN,$A0n,$A1n,$tao1n,$A2n,$tao2n,$A3n,$tao3n,$RateSusN;DelayUpdate	AppendToTable $Delayn,$commentn,$Fitstartn,$Fitendsn,$WeightingN		|******select a file*************FileName=DoOpenFileDialog ()print FileName|******Load sweeps from PatchMaster File*************					j=0	do		LoadPM/A=(gr_index[j])/B=(ser_index[j])/C=(sw_index[j])/D=2/N='Flash' FileName		j += 1	while(j<numpnts(gr_index))	j=0		do		// Main loop for this particular trace			CmN="Flash"+"_"+num2str(gr_index[j])+"_"+num2str(ser_index[j])+"_"+num2str(sw_index[j])+"_2_CM"			FitCmN="fit_"+CmN			TempFitCmN="tempfit_"+CmN			print "Processing ", CmN						Do				//Loop for fitting one trace - will run until user accepts fit as OK				$CmN[0]=Nan				display $CmN				showinfo									//Display Cm and let user choose the end of fitting range				WaveStats/Q $CmN				SetAxis left (V_avg-4*V_sdev), (V_avg+4*V_sdev)				InitialFitOKflag=0				FitEnd=FitEndSt									Do					SetAxis /A bottom					Cursor B, $CmN,FitEnd					DoUpdate					DoAlert 2, "Is cursor position OK?"								//allow user to see if its OK					if(V_Flag==1)												//advance if user says so						do						FitEnd+=0.1						while(numtype($CmN[FitEnd])!=0)					endif					if(V_Flag==2)												//go back is user says so						do						FitEnd-=0.1						while(numtype($CmN[FitEnd])!=0)					endif					while(V_Flag!=3)					FitEnd = xCsr(B)													WaveStats/Q/R=(0.0,0.45) $CmN				axislow=V_avg-3*V_sdev				WaveStats/Q/R=(0.55,0.60) $CmN				axishigh=V_max+2*V_sdev				SetAxis left (axislow), (axishigh)				setAxis bottom 0.45,0.75			//	setAxis bottom 4.45,4.75				ModifyGraph mode=4,marker=19,msize=1				ShowInfo									i=480	                                              				 //Let user choose the start of fitting range								Do                          Cursor/P A, $CmN,i				DoUpdate				DoAlert 2, "Is cursor position OK?"								//allow user to see if its OK				if(V_Flag==1)												//advance if user says yes					do						i+=1					while(numtype($CmN[i])!=0)				endif				if(V_Flag==2)												//go back if user says no					do						i-=1					while(numtype($CmN[i])!=0)				endif				while(V_Flag!=3)				t_start = xCsr(A)																					if(DBExp==3)																//Fit 3 exponentials				FitTripleE (CmN,"TriFitVar",FitEnd,t_start,0,weight)								$tao1n[ExPnts+j]=TriFitVar[2]; $tao2n[ExPnts+j]=TriFitVar[4]; $tao3n[ExPnts+j]=TriFitVar[6] 								$A1n[ExPnts+j]=-1*TriFitVar[1]; $A2n[ExPnts+j]=-1*TriFitVar[3]; $A3n[ExPnts+j]=-1*TriFitVar[5]				wavestats/Q/R=(0,FitStartSt) $CmN				print $CmN(FitStartSt+$tao2n[j]/2000)					//			if( ($tao2n[j]/$tao1n[j])<2  %|  ($tao1n[j]/$tao2n[j])<2 )						//if the two time constants are almost similar	//				print "Time constants to close together! Doing double exp fit instead"		//				FitTripleE (CmN,"TriFitVar",FitEnd,t_start,2)	//				$tao2n[j]=TriFitVar[4]; $tao3n[j]=TriFitVar[6] 					//			       $A2n[j]=-1*TriFitVar[3]; $A3n[j]=-1*TriFitVar[5]	//				$tao1n[j]=0.001														//				$A1n[j]=0	//				TriFitVar[1]=0	//				wavestats/Q/R=(0,0.49) $CmN	//			endif											endif						if(DBExp==2)										//Fit 2 exponentials								FitTripleE (CmN,"TriFitVar",FitEnd,t_start,2,weight)				 $tao2n[ExPnts+j]=TriFitVar[4]; $tao3n[ExPnts+j]=TriFitVar[6] 							       $A2n[ExPnts+j]=-1*TriFitVar[3]; $A3n[ExPnts+j]=-1*TriFitVar[5]				$tao1n[ExPnts+j]=0.1				$A1n[ExPnts+j]=0				TriFitVar[1]=0				wavestats/Q/R=(0,0.49) $CmN			endif						duplicate/O $CmN $TempFitCmN													//Calculate fit starting from cursor			$TempFitCmN= TriFitVar[0] + TriFitVar[1]*exp(-(x- TriFitVar[7])/ TriFitVar[2]) +  TriFitVar[3]*exp(-(x- TriFitVar[7])/ TriFitVar[4]) +  TriFitVar[5]*exp(-(x- TriFitVar[7])/ TriFitVar[6])			wavestats/Q/R=(0,FitStartSt) $CmN			$A0n[ExPnts+j]=V_avg			Findlevel/Q $TempFitCmN V_avg			//find crossing between fit and Cm(before)			if (V_flag==0)								//if a crossing was found				$DelayN[ExPnts+j]=V_levelX-FitStartSt			//use it to calculate the delay				t_startExtr=V_levelX					// - and the new starting point for the fit			else											//if no crossing was found				$DelayN[ExPnts+j]=Nan						//Delay is set to Nan				t_startExtr=t_start						// - and starting point set to left cursor			endif			Cm_start = TriFitVar[0]+TriFitVar[1]+TriFitVar[3]+TriFitVar[5]			print "Cm_before = ", $A0n[ExPnts+j]			print "Cm_start = ", Cm_start			print "DeltaCm = ", Cm_start-$A0n[ExPnts+j]			print "t_start = ", t_start			Dfit0=-(TriFitVar[1]/TriFitVar[2])-(TriFitVar[3]/TriFitVar[4])-(TriFitVar[5]/TriFitVar[6])	//Differential of fit at time 0			print "Dfit0= ", Dfit0			$A1n[ExPnts+j]=-1*TriFitVar[1]+(Cm_start-$A0n[ExPnts+j])*(-1*TriFitVar[1]/$tao1n[ExPnts+j])/Dfit0			//amplitudes corrected for missing capacitance			$A2n[ExPnts+j]=-1*TriFitVar[3]+(Cm_start-$A0n[ExPnts+j])*(-1*TriFitVar[3]/$tao2n[ExPnts+j])/Dfit0 			$A3n[ExPnts+j]=-1*TriFitVar[5]+(Cm_start-$A0n[ExPnts+j])*(-1*TriFitVar[5]/$tao3n[ExPnts+j])/Dfit0			print "TriFitVar[1] = ", -1*TriFitVar[1], "     A1n = ", $A1n[ExPnts+j]			print "TriFitVar[3] = ", -1*TriFitVar[3], "     A2n = ", $A2n[ExPnts+j]			print "TriFitVar[5] = ", -1*TriFitVar[5], "     A3n = ", $A3n[ExPnts+j]							duplicate/O/R=(0,FitEnd) $CmN $FitCmN															//Calculate fit starting from Cm before the flash			$FitCmN=$A0n[ExPnts+j]+$A1n[ExPnts+j]*(1-exp(-(x-t_startExtr)/$tao1n[ExPnts+j]))+$A2n[ExPnts+j]*(1-exp(-(x-t_startExtr)/$tao2n[ExPnts+j]))+$A3n[ExPnts+j]*(1-exp(-(x-t_startExtr)/$tao3n[ExPnts+j]))						i=trunc(x2pnt($CmN, t_startExtr)-1)				//set values of fit to Cm(before) if t<t_startExtr			$FitCmN[0,i]=$A0n[ExPnts+j]						if(DBExp==2)						$tao1n[ExPnts+j]=NaN			endif						grnam = winname(0,1)						//kills fit window			dowindow /k $grnam						display $CmN,$FitCmN			showinfo			ModifyGraph mode=0,lstyle($CmN)=1,rgb($CmN)=(0,0,0)				WaveStats/Q/R=(0.45,0.65) $CmN			SetAxis left axislow, axishigh			setAxis bottom 0.45,0.65			DoAlert 1, "Is initial fit OK?"				if (V_Flag==2) 					// Yes					InitialFitOKflag=1				endif					WaveStats/Q/R=(0.15,4) $CmN			SetAxis left ($A0n[ExPnts+j]-0.1e-12), (V_avg+3*V_sdev)			setAxis /A bottom			DoAlert 1, "Is overall fit OK?"				if (V_Flag==2) 					// Yes					InitialFitOKflag=1				endif		While (InitialFitOKflag==1)																						//Get capacitance for burst and sustained phase				$Amp1n[ExPnts+j]=$CmN(1.5)-$A0n[ExPnts+j]					//amplitude until 1 sec				$Amp2n[ExPnts+j]=$CmN(2.5)-$A0n[ExPnts+j]					//amplitude until 2 sec				$Amp4n[ExPnts+j]=$CmN(4.49)-$CmN(1.5)				//amplitude 1-4 sec slow comp.				$Fitstartn[ExPnts+j]=t_start				$Fitendsn[ExPnts+j]=FitEnd				$cellN[ExPnts+j]=gr_index[j]				$SeriesN[ExPnts+j]=ser_index[j]				$RateSusN[ExPnts+j]=($CmN(FitEndSt)-$A0n[ExPnts+j]-$A1n[ExPnts+j]-$A2n[ExPnts+j])/(FitEndSt-FitStartSt)				$FileNameN[ExPnts+j]=FileName				$WeightingN[ExPnts+j]=weight			//Let user see the final fit			WaveStats/Q/R=(0,4.5) $CmN			SetAxis left ($A0n[ExPnts+j]-0.1e-12), (V_avg+2.5*V_sdev)														SetAxis bottom 0,4.5			DoAlert 1, "Mark fit as Good?"			if(V_Flag==1)				$Commentn[ExPnts+j]="Good"			endif										if(V_Flag==2)				print fn_G			//	fn_G=fn+1			//	abort				$Commentn[ExPnts+j]="Bad"			endif			j+=1		print " j = ",j		while(j<(numpnts(gr_index)))endMacro FitTripleE(YName,ParName,t_end,t_start,Flag,weighting)	string YName,ParName	Variable t_end,t_start,flag,ii,weighting	//A triple exponential fit  to the curve on which the X-cursor sits	//A cursor has to be put onto a curve in the top window!	//ParName is the name of a wave which is created by the macro and which contains	// the fit parameters (called FitPar; see meaning of the parameters below )	// Fitting is done between cursor and t_end	//If flag is 2. the fastest component will be fixed to zero amplitude: 2 component fit	//otherwise  threecompnent fit	//  weights:  that  the first fifth of the  fitting range has 5 time the weigtht  as the rest 	// initial guesses:  A fixed pattern based on the x-range (Tot) and the y-range (Y_tot)	// of the data has been programmed (see below). It is readily changed		//FitPar[0] is the asymptotic Y-value; 	//FitPar[1],FitPar[2]  are amp and Tau  of fastest comp.	//FitPar[3],FitPar[4]  correspondingly of second compnent	//FitPar[5],FitPar[6]  correspondingly of third compnent	//FitPar[7]  is time 0, which is being fixed during the fitting		Variable  t_wave, Y_tot,tot	Make/N=8/D/O $ParName		Duplicate/O $YName, Weightf	Weightf = x	t_wave = Weightf[(numpnts(Weightf)-1)]		if  (t_end > t_wave)		t_end=t_wave		print "t_end changed to ",t_end	endif	tot = t_end - t_start	Y_tot = 1.5* ($YName (t_start) - $YName (t_end))		Weightf = 1	print "weighting",weighting	Weightf (t_start,t_start+tot/weighting)=weighting		print "tstart =", t_start	//initial guesses:	$ParName[0] = $YName (t_start) - Y_tot	$ParName[1] = 0.3*Y_tot 																$ParName[2] = 0.04	$ParName[3] = 0.5*Y_tot 	$ParName[4] =0.4	$ParName[5] =0.5*Y_tot 	$ParName[6] =tot	$ParName[7] =t_start	//Print $ParName[0]	If  (Flag ==2)		$ParName[1] = 0	//	Make/O/T/N=1 T_constraints	//	T_Constraints[0]={"K3>0"}		FuncFit/H="00100001"/L=10000 Triple $ParName $YName(t_start,t_end) /W=Weightf /D 					// chaged fixed t_start and y0		Print "2comp. Fit to:",  YName, "  from   ", t_start, "to  ", t_end,"sec"		Print  "Tau's:	", 1000*$ParName[4],1000*$ParName[6],"	msec"		Print  "Amps:	", -1e15*$ParName[3], -1e15*$ParName[5],"	fF"	ELSE		Make/O/T/N=5 T_Constraints		T_Constraints = {"K1 <0","K2 >0.001","K3 <0","K4 > 0.001","K5 <0"}		FuncFit/H="00000001"/L=10000 Triple $ParName $YName(t_start,t_end) /W=Weightf /C=T_Constraints /D				// chaged fixed t_start and y0		Print "3comp. Fit to:",  YName, "  from   ", t_start, "to  ", t_end,"sec"		Print  "Tau's:	", 1000*$ParName[2], 1000*$ParName[4],1000*$ParName[6],"	msec"		Print  "Amp's:	", -1e15*$ParName[1], -1e15*$ParName[3], -1e15*$ParName[5],"	fF"	EndifendFunction/D Triple (Parwave, x)	wave/D Parwave	Variable/D x	//Parwave[0] is final value; Parwave[1],Parwave[2]  are amp and timeconstant  of fastest comp.	//Parwave[3],Parwave[4]  correspondingly of second compnent	//Parwave[5],Parwave[6]  correspondingly of third compnent	//Parwave[7]  is time 0, which has to be fixed during the fitting		Variable result	result = Parwave[0] + Parwave[1]*exp(-(x-Parwave[7])/Parwave[2])	result  += Parwave[3]*exp(-(x-Parwave[7])/Parwave[4])	result  += Parwave[5]*exp(-(x-Parwave[7])/Parwave[6])	return resultendMacro Calc_Stats()variable tau	if(W_coef[2]<=W_coef[4])		tau=1/W_coef[4] 	else		tau=1/W_coef[2]	endif//		  tau=0.17825	print "tau=",tau	print "Component 1"	print 1000/W_coef[2]	print -1e15*W_coef[1]/exp(W_coef[2]*xcsr(A))	print "Component 2"	print 1000/W_coef[4]	print -1e15*W_coef[3]/exp(W_coef[4]*xcsr(A))	print "MaxRate"	WaveStats/Q/R=(0,0.19) Kcont1_1_1_1_Cm	print (Kcont1_1_1_1_Cm(xcsr(A)+tau/2)-V_avg)/(tau/2)	print Kcont1_1_1_1_Cm(xcsr(A)+tau/2)endmacroFunction/S DoOpenFileDialog ()	Variable refNum	string message = "Select a file"	string outputPath	String fileFilters = "Data Files (*.dat):.dat;"	fileFilters += "All Files:.*;"		Open /D /R /F=fileFilters /M=message refNum	outputPath = S_fileName	return outputPath	print outputPathend
